# The characteristics of caffeine intake in Croatian university students

**DOI:** 10.1093/eurpub/ckac131.233

**Published:** 2022-10-25

**Authors:** D Laslo, I Miskulin, N Srb, T Klepo, S Bonet, M Miskulin

**Affiliations:** Department of Public Health, Faculty of Medicine Osijek, University of Osijek, Osijek, Croatia

## Abstract

**Background:**

Excessive caffeine intake combined with increasing numbers and availability of caffeine-containing products in modern societies are causes for concern. University students may be at increased risk of excessive caffeine consumption due to seeking caffeinated products with well-known wakefulness effects and cognitive benefits. This study aimed to highlight the characteristics of caffeine consumption among Croatian university students.

**Methods:**

This cross-sectional questionnaire study was conducted from May 2020 to April 2021 period. A validated, anonymous questionnaire that contained questions regarding demographic data, data about caffeine consumption habits, and its sources was self-administered via an online link to a cross-faculty representative student sample of the University of Osijek in Eastern Croatia.

**Results:**

The study sample included 1197 subjects with, median age of 22 years (interquartile range 21-24), 24.1% males, and 75.9% females. The median caffeine intake was 512.0 mg/day (interquartile range 228.0-972.0). The higher caffeine intake was observed in part-time students (p = 0.026), students who were preparing for exams during the participation in this study (p = 0.010), and students who smoked cigarettes (p < 0.001). There was a poor positive correlation between the amount of caffeine intake and academic success connected with caffeine consumption (rs = 0.225; p < 0.001). There were fair positive correlations between the amount of caffeine intake and caffeine consumption because of the avoidance of the withdrawal symptoms (rs = 0.490; p < 0.001) and between the amount of caffeine intake and knowledge of the impact of caffeine consumption on the occurrence of social conflicts (rs = 0.349; p < 0.001).

**Conclusions:**

The study revealed high caffeine intake among Croatian university students. The implementation of specific preventive measures directed toward the protection of students’ health from adverse health effects related to caffeine is needed.

**Key messages:**

• Croatian university students have displayed high caffeine intake in their everyday life.

• There is a need for the implementation of specific preventive measures directed toward the protection of students’ health from adverse health effects related to caffeine consumption.

